# PRIME-nurse practitioner competency model validation and criterion based OSCE rubric interrater reliability

**DOI:** 10.1186/s12909-024-05056-3

**Published:** 2024-02-07

**Authors:** Rita D’Aoust, Sarah E. Slone, Nancy Russell, Chakra Budhathoki, Catherine Ling

**Affiliations:** 1 Johns Hopkins School of Nursing, Baltimore, MD USA; 2 Johns Hopkins School of Medicine, Baltimore, MD USA

**Keywords:** RIME, PRIME-NP, Competency, Validity, Reliability, Nurse practitioner

## Abstract

The PRIME-NP (Professional-Reporter-Interpreter-Manager-Educator/Evaluation-Nurse Practitioner) Model is adapted from the RIME (Reporter-Interpreter-Manager-Educator) model used in medical education to guide medical student and resident education. The Delphi technique was used to validate the PRIME-NP Model. After two rounds of review by a group of experts in NP curriculum, the model was determined to be valid based on expert consensus. Agreement percent increase from the first round to the second round in all categories. Interrater reliability (IRR) was assessed using interclass correlation after instrument validation was completed for each of the five levels of the PRIME-NP model. Overall, the IRR of the instrument was found to be acceptable with some notable exceptions. No variance was noted in professional behaviors at any level. Variance was increased in management and educator/evaluator behaviors in higher/later course levels. The PRIME-NP Model and PRIME-NP OSCE Rubric is a valid and reliable instrument to assess NP student progression in objective structured clinical examinations. This instrument has the potential for adaptation for use in other types of health sciences education and settings.

The PRIME-NP Model is adapted from the RIME (Reporter-Interpreter-Manager-Educator) model used in medical education to guide medical student and resident education [1, 2]. PRIME-NP is a mnemonic for Professional-Reporter-Interpreter-Manager-Educator/Evaluation, the five integral functional areas that nurse practitioners (NPs) must competently perform to evaluate, treat, and manage patient health needs [3]. The PRIME-NP framework is a competency-based approach to teach and assess nurse practitioner students’ development into competent providers. Students are evaluated on a level-specific continuum on each of the five performance levels: professional, reporter, interpreter, manager, and educator/evaluator with expected achievement of mastery of all five levels upon graduation and readiness for entry to practice.

## Background

Pangaro first described the RIME model in 1999 as a developmental framework for assessing medical learners’ progression and achievement of competency in clinical settings [1]. RIME is a mnemonic for Reporter-Interpreter-Manager-Educator. As students advance in their knowledge, skills, and attitudes, they typically go through four developmental stages: Reporter, Interpreter, Manager, and Educator. Each stage involves the integration of knowledge, skills, and attitudes, with more advanced stages requiring greater sophistication and confidence. The characteristics of each stage are outlined as follows:

Reporter: Reporters proficiently gather clinical information for their patients, demonstrating clear communication both verbally and in writing. They can elicit and discern important information, focus on central issues, and provide a cogent presentation of the findings.

Interpreter: Interpreters independently identify and prioritize problems, developing a differential diagnosis for a patient’s central problem(s).

Manager: Managers formulate and defend diagnostic and therapeutic plans for their patients’ central problem(s). They use clinical judgment to determine when action is necessary and analyze the risk/benefit balance based on individual patient circumstances.

Educator: Educators master fundamental skills and possess insight to define important research questions, seek evidence behind clinical practice, and critically evaluate its quality. Educators actively contribute to educating the team.

The RIME Model was later adapted for use by some as the PRIME model in which the professional aspects integrated in each RIME area was abstracted as a separate and explicit category. Assessment and validation of physician clinical knowledge and competency are also components of the United States Medical Licensing Examination (USMLE) Step 2 and Step 3 examination [4]. Clinical skills knowledge are specifically assessed through USMLE Step 2 Part 1 Clinical Knowledge and Step 2 Part 2 Clinical Skills. The USMLE Step 2 Clinical Skills is determined to be crucial for the enhancement of skills vital to supervised and unsupervised practice of medical students [5]. However, convenience and cost to students remain as significant barriers to continued use for Part 2 Clinical Skills exam [6]. Despite these barriers, clinical competency validation continues to be critical in ensuring public accountability for competence [5]. USMLE Step 2 Clinical Skills was halted during COVID. Rather than relaunching the current format for Step 2 Clinical Skills, USMLE is expected to continue the assessment of physician clinical competency, but in a different format that preserves public accountability for clinical skills assessment while improving access and decreasing costs [7].

NP student clinical encounters as well as their clinical progress are tracked and evaluated by faculty members. Incorporating the use of standardized patients coupled with objective structured clinical exams that reflect a curricular model of competency progression (blueprint) should guide and maximize the strengths of student clinical evaluations to ensure consistent, objective, and authentic clinical competency performance [8].

Like other health care professions, nursing has recently begun to move to competency-based education through the adoption of a new transformational competency-based model and framework including core competencies for nurse practitioner education [9–11]. In addition to this transition, a recent report of the National Task Force Criteria for Evaluation of Nurse Practitioner Programs, Standards for Quality Nurse Practitioner Education added a focus on individual level competency assessment through formative and summative evaluation [10]. The PRIME-NP Model for clinical skills development and assessment aligns with these current and emerging standards in education.

Nurse practitioner (NP) programs are expected to prepare NPs for full scope of practice in ever-evolving, highly complex, and fast-paced clinical environments upon graduation. However, clear and consistent definitions regarding what constitutes clinical competency and how to reliably assess competency remain nebulous [12, 13]. Clinical competency performance is assessed in a variety of didactic, clinical, and simulation settings, and therefore, it is imperative that any assessment instrument must be easily understood by students, faculty, and preceptors and be relevant to multiple settings. The model and instrument should objectively explicate and capture a student’s progress toward meeting NP competencies while also ensuring that the student evaluation is consistent across evaluators. Objective Structured Clinical Exams (OSCEs) assessments are structured to minimize bias and subjectivity while aligning to expected performance behaviors. OSCEs provide realistic clinical practice to assess multiple competencies in which health providers such as NPs apply theoretical knowledge that mirror challenges seen in actual practice [14].

Practice-based assessments can be fraught with numerous threats to validity and reliability [15]. NP clinical competency assessment must be grounded in evidence. An instrument must be validated prior to adoption to ensure that the instrument provides a consistent objective measure of clinical competency performance. This paper describes the model validation and measurement of interrater reliability of the PRIME-NP model to support the model development and use previously reported [3].

## Methods

Pangaro’s PRIME Model was adapted for NP clinical competency-based education (PRIME-NP) and previously described in the literature [1, 3, 16]. The five PRIME-NP Model categories (Professional Behaviors, Reporter, Interpreter, Manager, and Evaluator/Educator) were selected to reflect the progression of the student’s growth in knowledge, skills and attitudes throughout their clinical courses. The five selected categories were mapped onto the clinical curriculum at our institution to reflect when they should be introduced, practiced and finally mastered. A rubric with expected performance behaviors was then developed based on these five categories as an instrument assess student performance in Objective Structured Clinical Examination (OSCE) encounters. The template of the rubric is consistent across the five clinical courses, but the point weights, details, and expectations successively build along with the student’s progression through the program. The PRIME-NP OSCE Rubric is applied through clinical scenarios in which the expected performance level is described for the applied scenario. The purpose of this project was to validate the PRIME-NP evaluation model and assess the inter-rater reliability (IRR) for the model. The study was approved by the ethics committee of Johns Hopkins University (IRB00322391 and IRB00335668). The procedures used in this study adhere to the tenets of the Declaration of Helsinki.

### Validation of PRIME-NP model, indicators, and performance rubric progression using Delphi technique

The Delphi Technique is a systematic and structured method of developing consensus among expert panel members through a series of iterative questionnaires with controlled feedback, statistical group response, and anonymous expert input. The Delphi Technique can be an especially useful research methodology when there is no true or knowable answer, such as decision-making, policy, or long-range forecasting. A wide range of expert opinions can be included, which can be useful in cases in which relying on a single expert would lead to bias. In healthcare, the Delphi process had been used to evaluate current, resolving controversy in management, formulate theoretical or methodological guidelines, develop assessment instruments and indicators, and formulate recommendations for action and prioritizing measures [17].

**Fig. 1:**
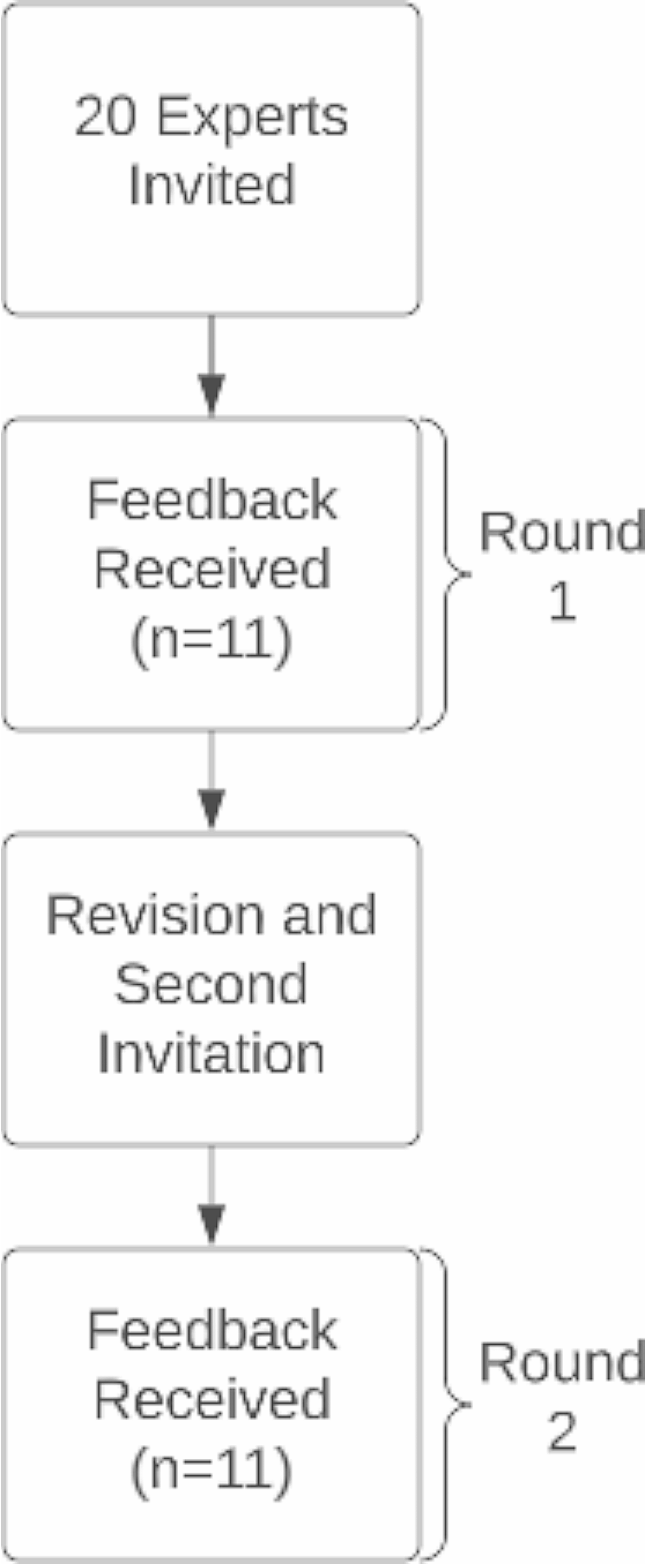
Development of validated PRIME-NP model using the Delphi technique

The PRIME-NP Model for Clinical Competency Development and Assessment was adapted and developed for Nurse Practitioner student education, but the model validation and interrater reliability was not reported and is the focus of this paper [3]. For the purpose of adaptation, a group of experts/reviewers in NP curriculum was approached to provide consensus feedback on the PRIME-NP Model, indicators for each area, and expected performance levels within PRIME-NP across courses (curriculum roadmap rubric). A total of 20 national nursing subject matter experts were invited to participate in two rounds of Delphi surveys using Qualtrics survey instrument (Fig. 1). Expert faculty were asked a series of questions to assess expert consensus for the model, indicators, and progression:

1. Is the PRIME model adaptation to Advanced Practice Nurse Practitioner an appropriate fit?;
2. For each PRIME category, do the indicators adequate reflect (measure) the category and do the indicators with descriptors, adequately measure the indicator?;
3. Do the point allocation reflect the right distribution within a category?; and.
4. Do the point allocation for each PRIME category appropriate to reflect progressions from Level I to Level V?

Reviewer agreement was determined for each component and item of the OSCE rubric. A free text comment field associated with each item was also included to collect feedback if they did not agree with the item or if they had any additional comments.

### Assessment of inter-rater reliability (IRR)

**Fig. 2:**
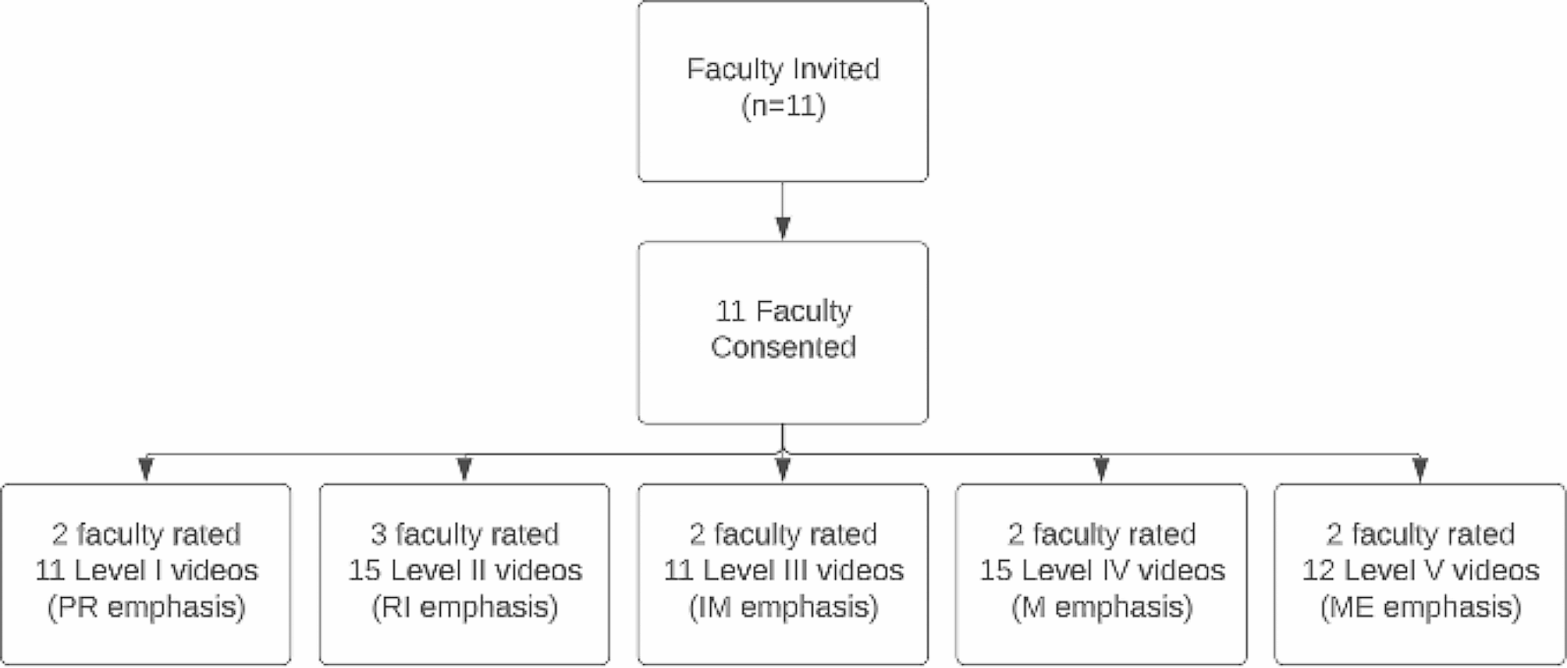
Faculty recruitment and participation in IRR assessment *Note*: All ratings were conducted independently

IRR testing was the second part of the revised instrument evaluation process. For an overview of the process, please see Fig. 2.

Trained OSCE faculty observers from within a school of nursing were recruited to participate in IRR of the validated PRIME-NP instrument. Faculty participants inclusion criteria were greater than 21 years of age, English speaking, and licensed and certified as an NP in Adult-Gerontology, Family, or Pediatric Primary Care. Faculty were offered a $40 stipend per OSCE case reviewed, but no more than $600 for any one faculty. Faculty members were recruited via email by a research team member consistent with the methods described in the approved research protocol. Informed consent was obtained from all the participants. After consent was obtained, written instructions were provided and a meeting to review the instrument was held.

Each participant was sent 11–15 student OSCE video recordings of a clinical level case and the associated PRIME-NP Model rubric via a secure OneDrive that only a single faculty participant and the study team could access. Participants were blinded to the student identities although some faculty may recognize a student in the videotape. Each participant rated the student performance using the PRIME-NP rubric independently. All five clinical levels were evaluated by two to three raters and each level used a unique clinical scenario (see Fig. 2).

Intraclass correlation (ICC) was then determined for each clinical level to evaluate IRR. This data analytic method was selected as it is a more widely acceptable method to determine IRR for quantitative scores than a simple description of inter-rater agreement as there will innately be some variance between scores for the same scenario given the variability of student performance, standardized patient interpretation of the scenario, and faculty rater expectations [18]. The instrument evaluation utilized a fully crossed design in which each faculty rater provided ratings for every unit of assessment for the PRIME-NP rubric and independent of other faculty evaluators. The lack of variation in inter-rater agreement when assessing professional behaviours is unsurprising as these behaviours are cultivated in pre-licensure programs but nonetheless, may account for potential biases [19]. All data were de-identified prior to analysis and analyzed using IBM SPSS Statistics (Version 27).

## Results

### Delphi results

The distribution of expertise among the reviewers is provided in Table 1.

**Table 1:** Distribution of experts that responded to the Delphi survey

Field	First Round, n (%) (*n* = 11)	Second Round, n (%) (*n* = 11)
1. APRN	6 (54.5%)	7 (63.6%)
2. Simulation	4 (36.4%)	4 (36.4%)
3. Curricular design	6 (54.5%)	8 (72.7%)
4. Program administration	4 (36.4%)	6 (54.5%)
5. Faculty	6 (54.5%)	9 (81.8%)

Note: respondents were allowed to select more than one area of expertise

Eleven out of 20 invited reviewers completed the survey for a response rate of 55% in both rounds. The survey responses were anonymous, so it is not known if the same reviewers responded in each round. Therefore, data were summarized by round only rather than performing a matched analysis. Only the available data were summarized, as there were some item-level and/or category level missing responses. The Case Presentation item under Reporter category in both Delphi surveys was not answered by respondents. Table 2 summarizes the agreement frequency and percent for each response by each PRIME-NP item.

**Table 2:** Summary of PRIME-NP category clinical indicator agreement

Professional category Items, n (%)	First Round (*n* = 11)	Second Round (*n* = 1)
1. Washes hands before beginning examination	6 (85.7%)	9 (90.0%)
2. Displays a professional demeanor and interacts appropriately with the patient	7 (100.0%)	10 (100.0%)
3. Demonstrates humanistic qualities– (Quantified with SP Communication)	7 (100.0%)	10 (100.0%)
**Reporter** category Items, n (%)	First Round (*n* = 8)	Second Round (*n* = 11)
1. Chief complaint	8 (100.0%)	11 (100.0%)
2. HPI	8 (100.0%)	11 (100.0%)
3. Health history	8 (100.0%)	11 (100.0%)
4. Review of systems	8 (100.0%)	11 (100.0%)
5. Physical Exam	8 (100.0%)	11 (100.0%)
**Interpreter**, Interpreter indicators, n (%)	First Round (*n* = 11)	Second Round (*n* = 11)
1. Articulate appropriate differential diagnoses	6 (85.7%)	9 (90.0%)
2. Choose, justify and correctly interpret diagnostic testing	6 (100.0%)	9 (100.0%)
3. Provide Correct Diagnosis	6 (100.0%)	9 (100.0%)
**Manager**, Manager indicators, n (%)	First Round (*n* = 11)	Second Round (*n* = 11)
1. Develops a complete plan of care appropriate for the actual diagnosis and baseline medical conditions	6 (85.7%)	9 (90.0%)
2. Uses shared decision-making to develop diagnostic and treatment plan with follow up options with pt	6 (100.0%)	9 (100.0%)
3. Provides age-appropriate screening recommendations	6 (100.0%)	9 (100.0%)
4. Able to state where/how applying specific Clinical Guidelines	6 (100.0%)	9 (100.0%)
5. Discusses Collaborative/Team-based care	6 (100.0%)	9 (100.0%)
**Educator/Evaluator**, Educator/Evaluator indicators, n (%)	First Round (*n* = 11)	Second Round (*n* = 11)
1. Provides tailored education about components of treatment and testing with rationale	7 (100.0%)	10 (100.0%)
2. Provides Appropriate referral instruction	7 (100.0%)	10 (100.0%)
3. Utilizes behavior supporting communication	7 (100.0%)	10 (100.0%)
4. Provides Case specific Follow Up	7 (100.0%)	10 (100.0%)

Note: Not all participants responded to all questions

Although 11 content experts responded to the Delphi survey, the number responding to each category varied as a response to each question was not forced in the survey. Within each PRIME-NP category agreement varied on the first round. In the professional category, item agreement percent ranged from 85.7 to 100% in the first round. Agreement ranged from 90.0 to 100% in this category in the second round.

There are three sub-areas for the Reporter category: health history, physical exam, and case presentation. The Reporter summary for each sub-category is presented although detailed indicators for each sub-category is available. For Reporter Health History sub-category, agreement ranged from 75 to 100% in the first round, and it ranged from 81.8 to 100% in second round. For the Reporter Review of Systems category, agreement ranged from 87.5 to 100% in the first round, and it was 100% for all items in the second round. For the Reporter Physical Exam sub-category, agreement ranged from 87.5 to 100% in first round, and it ranged from 90.9 to 100% in second round. In the interpreter category, agreement ranged from 85.7 to 100% in the first round, and from 90.0 to 100% in the second round. In the manager category, agreement ranged from 85.7 to 100% in the first round, and it ranged from 90.0 to 100% in the second round. Educator/Evaluator category agreement was 100% for all items in both rounds.

Expected performance within a category for a specific level and progression category revealed a high consensus (Table 3).

**Table 3:** Assessment weighting within a category and progression and model fit

Weighting of indicators within a category and progression across levels	First Round (*n* = 11)	Second Round (*n* = 11)
**Right point allocation within a category? n (%)**		
1. Professional	5 (100.0%)	7 (87.5%)
2. Reporter	5 (100.0%)	8 (100.0%)
3. Interpreter	5 (100.0%)	8 (100.0%)
4. Manager	5 (100.0%)	8 (100.0%)
5. Educator/Evaluator	5 (100.0%)	8 (100.0%)
**Right point allocation within a category to reflect progression from novice to competent?** n (%)		
1. Professional	5 (100.0%)	7 (87.5%)
2. Reporter	5 (100.0%)	8 (100.0%)
3. Interpreter	5 (100.0%)	7 (87.5%)
4. Manager	5 (100.0%)	7 (87.5%)
5. Educator/Evaluator	5 (100.0%)	7 (87.5%)
I**s PRIME model adaptation to APN practitioner evaluation appropriate?** n (%)	First Round	Second Round
1. Professional	6 (85.7%)	9 (90.0%)
2. Reporter	7 (100.0%)	10 (100.0%)
3. Interpreter	7 (100.0%)	10 (100.0%)
4. Manager	7 (100.0%)	10 (100.0%)
5. Educator/Evaluator	6 (100.0%)	9 (100.0%)

Note: Not all participants responded to all questions

In terms of point allocation within a category, there was 100% agreement in the first round, and agreement ranged from 87.5 to 100% in the second round. In terms of point allocation within a category that would reflect progression from novice to competent level, there was 100% agreement in the first round, and agreement ranged from 87.5 to 100% in the second round. When asked if the PRIME model adaptation to APN practitioner evaluation was appropriate, the agreement ranged from 85.7 to 100% by competency level in the first round. Similarly, the agreement ranged from 90.0 to 100% in the second round.

### IRR results

The analysis results of IRR are presented in Table 4.

**Table 4:** Assessment of inter-rater reliability using intra-correlation coefficient and associated statistics for five PRIME categories

Clinical Level	Professional	Reporter	Interpreter	Manager	Educator/ Evaluator
1					
ICC (*p*-value)	*	-0.32 (0.846)	*	0.00 (0.500)	*
95% CI		[-0.76, 0.31]		[-0.58, 0.58]	
2					
ICC (*p*-value)	*	0.14 (0.184)	0.06 (0.362)	0.25 (0.059)	0.43 (0.004)
95% CI		[-0.14, 0.51]	[-0.26, 0.56]	[-0.06, 0.60]	[0.11, 0.72]
3					
ICC (*p*-value)	*	0.26 (0.212)	0.44 (0.079)	0.28 (0.190)	-0.28 (0.810)
95% CI		[-0.38, 0.73]	[-0.19, 0.81]	[-0.35, 0.74]	[-0.74, 0.35]
4					
ICC (*p*-value)	0.33 (0.020)	0.43 (0.004)	0.12 (0.219)	0.32 (0.022)	0.06 (0.341)
95% CI	[0.01, 0.66]	[0.11, 0.73]	[-0.16, 0.49]	[0.01, 0.66]	[-0.20, 0.43]
5					
ICC (*p*-value)	0.65 (0.009)	0.70 (0.004)	0.22 (0.232)	0.75 (0.002)	0.03 (0.468)
95% CI	[0.14, 0.88]	(0.23, 0.90)	[-0.38, 0.69]	[0.34, 0.92]	[-0.56, 0.59]

* Both raters assigned the same maximum score to all videos/students, so no variance and ICC cannot be estimated

The estimated IRR values can be categorized using the Cicchetti method explained as: [1] < 0.40 poor; 0.40–0.59 fair; 0.60–0.74 good; 0.75-1.00 excellent [20]. Based on the ICC cutoff values, clinical level 5 showed “good” IRR for professional and reporter categories, and “excellent” IRR for manager category. For clinical level 4, only reporter category showed “fair” IRR. Similarly, in clinical levels 3 and 2, only interpreter and educator/evaluator categories, respectively showed “fair” IRR. No variance was noted in Professional at any level. Variance was increased in Management and Educator/Evaluator behaviors in higher/later course levels.

## Discussion

While the PRIME-NP Model development and use was previously described by D’Aoust and colleagues, the validation and reliability testing described in this paper provide scientific merit for its use [3]. The results of the Delphi survey indicate that there is high validity of the adapted PRIME-NP model rubrics after two rounds. The agreement percentage increased from the first round to the second round overall for the instrument. In the second round, all agreement percent estimates were above 80%. This indicates that the adapted PRIME-NP model, indicators, and rubric for performance within a level and progressive weighting is valid for NP student evaluation. Not conducting a matched analysis of survey responses between the 1st and 2nd rounds in the Delphi consensus method may impact validity. This limitation impedes tracking individual expert opinions over time, assessing changes in consensus, evaluating reliability and consistency, controlling for confounding factors, understanding the dynamics of influential experts, and maintaining sensitivity to subtle opinion changes. A matched analysis contributes to the reliability and validity of the Delphi process, offering valuable insights into how expert opinions evolve and the dynamics of consensus-building.

It was necessary to evaluate the IRR of the instrument to ensure faculty ratings using the instrument were reliable and consistent. The purpose of the IRR was to determine the extent of variability in faculty rating of student performance using the revised instrument. Overall, the IRR of the instrument was found to be acceptable with some notable exceptions (See Table 4). It is not notable that there is no variation in inter-rater agreement in the assessment of professional behaviors as these behaviors are developed in pre-licensure programs. However, there is significant variance among the faculty ratings in the management and educator/evaluator domains, as the complexity of the assessment increases. This increase is expected, as IRR is known to decrease as the complexity of the assessment increases [21].

Training of the raters included small group training on the PRIME-NP model and the use of the instrument itself including the criteria per level. However, specific training was not provided on the cases used to establish IRR. The training focused solely on the instrument and not the cases may account for some of the variability in ratings as professional behavior expectations are consistent, but the expected performances of the behaviors in the case are not consistent. As previously noted, variance was increased in Management and Educator/Evaluator behaviors in higher/later course levels. These differences reflect a need for more explicit expectations to be built into the instrument and refined faculty training.

Although the PRIME-NP Model was found to be reliable and valid for evaluation of NP student progression, certain limitations exist. The instrument was validated by a diverse group of experts from across the United States. However, the lack of recruitment of and validation by international and other healthcare experts raises whether results would have differed if a broader sample of experts had been recruited. Assessment of reliability of the instrument was conducted in a single school of nursing. There may be factors outside the PRIME-NP Model training that impacted IRR such as faculty onboarding training and selection bias. Not conducting a matched analysis between IRR survey responses in different rounds can compromise the validity calculation by limiting the ability to make individual-level comparisons, increasing variability, and potentially confounding the interpretation of changes observed over time.

The potential recognition of students in videotapes by faculty members can introduce a range of impacts on the expected performances of behaviors, especially when there are inconsistencies in how these behaviors are demonstrated. Faculty members who recognize students in videotapes may unintentionally introduce observer bias. Knowing the identity of the student may influence their expectations or evaluations, leading to a subjective assessment that may not accurately reflect the student’s actual performance. Faculty members serving as inter-raters may unconsciously attribute positive traits or overlook shortcomings based on their previous knowledge of the student, impacting the fairness and objectivity of the assessment. In addition, there can be decreased reliability as student recognition by some faculty evaluators influence ratings for recognized students but not others.

## Conclusions

The PRIME-NP Model is a valid and reliable instrument to assess NP student progression. Accurate assessment of NP progression is vital to ensuring competency of NP to practice upon graduation. Demonstrating both validity and reliability, this model provides a comprehensive and accurate means of assessing the developmental journey of NP students. The significance of objectively and accurately assessing NP student progression cannot be overstated, as it plays a pivotal role in guaranteeing the competency of NP graduates as they enter professional practice.

Ensuring that NP students are advancing appropriately in their education and clinical skills is paramount for producing highly competent and qualified healthcare professionals. The PRIME-NP Model, with its proven validity and reliability, serves as a tested assessment model and instrument in this endeavor. By offering a systematic and standardized approach to assessing NP student progression, it enhances the overall quality of education and contributes to the preparation of graduates who meet the rigorous demands of contemporary healthcare.

The versatility and effectiveness of the PRIME-NP Model suggest that it holds the potential for widespread adoption by schools of nursing. Its applicability extends beyond nursing education, as the principles and methodologies embedded in the model could conceivably be adapted for use by other health professions. This adaptability underscores the instrument’s utility in fostering standardized assessment practices across various healthcare disciplines, thereby contributing to a more unified and comprehensive approach to evaluating student progress and ensuring the competence of future healthcare professionals. As institutions strive for excellence in healthcare education, the PRIME-NP Model emerges as a valuable asset with the capacity to enhance assessment practices and, consequently, the overall quality of healthcare practitioners entering the workforce.

Next steps include development and piloting of formalized training on how to assess student performance in specific OSCE scenarios and determining the impact on IRR. Additional studies will also examine replicating the study with other NP program tracks in our curriculum and exploring this model’s utility in virtual reality (VR), augmented reality and mixed reality OSCEs. Future adaptations of PRIME-NP model include adapting the model for use in telehealth encounters and use of evaluation of NP competency in practice.

## Data Availability

The dataset generated and analyzed during the current study is available from the corresponding author on reasonable request.

## References

[1] Pangaro L (1999). A new vocabulary and other innovations for improving descriptive in-training evaluations. Acad Med.

[2] Ogburn T, Espey E (2003). The R-I-M-E method for evaluation of medical students on an obstetrics and gynecology clerkship. Am J Obstet Gynecol.

[3] D’Aoust RF, Brown KM, McIltrot K, Adamji J-MD, Johnson H, Seibert DC (2022). A competency roadmap for advanced practice nursing education using PRIME-NP. Nurs Outlook.

[4] Jeyaraju M, Linford H, Bosco Mendes T, Caufield-Noll C, Tackett S. Factors leading to successful performance on U.S. National Licensure exams for Medical students: a scoping review. Acad Med. 2023;98(1). 10.1097/ACM.0000000000004877 35857389

[5] Haist SA, Butler AP, Paniagua MA (2017). Testing and evaluation: the present and future of the assessment of medical professionals. Adv Physiol Educ.

[6] Flier LA, Henderson CR, Treasure CL (2016). Time to eliminate the step 2 clinical skills examination for US Medical graduates. JAMA Intern Med.

[7] Exams USML. Work to relaunch USMLE Step 2 CS discontinued. 2021.

[8] Bierer SB (2016). Handbook on Medical Student evaluation and Assessment. Teach Learn Med.

[9] American Association of Colleges of Nursing. The Essentials: Core competencies for professional nursing education. https://www.aacnnursing.org/Portals/0/PDFs/Publications/Essentials-2021.pdf: Retrieved November 12, 2022.

[10] National Organization of Nurse Practitioner Faculty. Standards for Quality Nurse Practitioner Education: A Report of the National Task Force on Quality Nurse Practitioner Education, 2021.

[11] Thomas A, Crabtree MK, Delaney K, et al. Nurse practitioner core competencies content. The National Organization of Nurse Practitioner Faculties; 2017.

[12] Lejonqvist G-B, Eriksson K, Meretoja R (2012). Evidence of clinical competence. Scand J Caring Sci.

[13] Nabizadeh-Gharghozar Z, Alavi NM, Ajorpaz NM (2021). Clinical competence in nursing: a hybrid concept analysis. Nurse Educ Today.

[14] Pangaro LN, McGaghie WC, editors. Handbook on medical student evaluation and assessment. Gegensatz Press; 2015. Jul 17.

[15] Williams RG, Klamen DA, McGaghie WC (2003). SPECIAL ARTICLE: cognitive, social and environmental sources of Bias in clinical performance ratings. Teach Learn Med.

[16] Pangaro L, Ten Cate O (2013). Frameworks for learner assessment in medicine: AMEE Guide No. 78. Med Teach.

[17] Nasa P, Jain R, Juneja D (2021). Delphi methodology in healthcare research: how to decide its appropriateness. World J Methodol.

[18] Gisev N, Bell JS, Chen TF (2013). Interrater agreement and interrater reliability: key concepts, approaches, and applications. Res Social Administrative Pharm.

[19] Hallgren KA (2012). Computing Inter-rater Reliability for Observational Data: an overview and Tutorial. Tutorials in Quantitative Methods for Psychology.

[20] Cicchetti DV (1994). Multiple comparison methods: establishing guidelines for their valid application in neuropsychological research. J Clin Exp Neuropsychol.

[21] Gwet K. Handbook of inter-rater reliability: the definitive guide to measuring the extent of agreement among raters. Advanced Analytics, LLC; 2014.

